# Research on Current Curative Expenditure among Lung Cancer Patients Based on the “System of Health Accounts 2011”: Insights into Influencing Factors

**DOI:** 10.7150/jca.34891

**Published:** 2019-10-20

**Authors:** Shuang Zang, Huan Zhan, Liangrong Zhou, Xin Wang

**Affiliations:** 1 School of Nursing, China Medical University, Shenyang, Liaoning;; 2 College of the Humanities and Social Sciences, China Medical University, Shenyang, Liaoning;; 3 School of Humanities and Management, Hunan University of Chinese Medicine, Changsha, Hunan.

**Keywords:** lung cancer, current curative expenditure, System of Health Accounts 2011

## Abstract

Purpose: To investigate the total current curative expenditure (CCE) of lung cancer in Hunan Province, China under the framework of the System of Health Accounts 2011 (SHA 2011) and explore the effect of insurance status, surgery and length of stay on the hospitalization expenses of patients with lung cancer.

Methods: Through multistage stratified cluster random sampling, a total of 46,214 patients with lung cancer were enrolled from 1,072 medical institutions in Hunan Province in 2016. Under the SHA 2011 framework, the lung cancer CCE was analyzed. The relationships between hospitalization expenditure and the following factors (surgery, type of hospital, insurance status, length of stay, institution level, age and sex) were analyzed using Spearman's correlation analyses, and how these factors influenced hospital expenditure was explored through multiple stepwise regression analysis and structural equation modelling.

Results: The CCE for lung cancer patients was 8063.75 million CNY. In total, 96.03% of the CCE for lung cancer occurred in hospitals and 58.88% of the expenditure flowed to general hospitals. The highest expenditures were incurred in the group aged 55-74 y, which accounted for 61.58% of the CCE. Drugs accounted for the greatest share expenditure to lung cancer patients at 34.31% of the CCE. Surgery, insurance status, institution level, sex and hospital type explained 57.5% of the variance in hospital expenses. The hospitalization expenses were related to surgery, insurance status, institution level and sex (rs = 0.033-0.688, p < 0.001). Surgery, insurance status and length of stay had direct effects on hospitalization expenses. Length of stay mediated the relationship between surgery and hospitalization expenses for lung cancer patients. Surgery mediated the relationship between insurance status and hospitalization expenses. All of these variables can explain 45% of the variance in hospitalization expenses.

Conclusions: The CCE of lung cancer is extremely high. The problems related to treatment efficiency and equity are serious for lung cancer patients in China. It is essential to expand health insurance coverage and reduce the curative expenditure of lung cancer.

## Introduction

Lung cancer is the most common malignant tumor in the world and the leading cause of cancer death. It is estimated that 2.1 million new cases of lung cancer and 1.8 million deaths from lung cancer have been reported in China, accounting for 37.0% of all new cancer cases and 39.2% of all cancer-related deaths 1. Moreover, lung cancer is the most costly type of malignant tumor 2. Once diagnosed, patients need to undergo intensive medical treatment and aggressive care, which leads to substantial increases in curative expenditure. More accurate diagnostic techniques and treatments have been developed and applied in the clinical practice of managing lung cancer 3,4. Although novel and advanced cancer therapeutic treatments have improved the life expectancy and clinical outcomes, these treatments consequently lead to a rising amount of expenditure for lung cancer patients 5. In China, the per capita medical expenditure for lung cancer patients has climbed from 40,508 CNY in 2005 to 66,020 CNY in 2014 6. The high expenditure for lung cancer may create great challenges for government, families and patients.

Studies on lung cancer expenditure and its influencing factors have been carried out globally. The data for the studies on lung cancer expenditure mainly came from the Medicare database 7 and medical institution records 8. As most of the research data were roughly estimated from medical records, the expenditures, such as fixed assets that were not used on patients, were still included in the real expenditures on health care. The actual final consumption of goods and services purchased on behalf of the individuals cannot be calculated clearly. For researchers to accurately account for the curative expenditure of lung cancer, there is a pressing need to establish an expenditure account framework that both increases the accuracy of the health accounts and enables broader comparability across countries. In recent years, with significant gains in economic strength and financial revenue, the state financial allocation used for public health has jumped annually. However, there is still a significant gap between the health services provided and public demand, and the problem of “high costs to receive treatment” is still grim 9. How serious is the situation? What are the monetary flows for expenditure on lung cancer? What are the major influencing factors of current care expenditure according to the beneficiary characteristics? All of these questions can be answered under the System of Health Accounts 2011 (SHA 2011) framework.

SHA 2011 is a standard framework that can help ensure the best quality of patients' expenditure information by institutionalizing deals for data collection, embedding the data in the information system, enforcing data production, and promoting data use. The SHA 2011 framework is now considered the global standard for the construction of national health accounts and has been adopted by all member countries of the European Union, nearly all member countries of the Organization of Economic Cooperation and Development and many additional countries 10. Under the SHA 2011 framework, “current expenditure on health care” and “gross capital formation in health care” have been separated. The SHA 2011 framework abandons the expression of total health expenditure, although it recommends the usage of current health expenditure, which refers to the final consumption of health care goods and services by the government, nonprofit institutions and households, excluding the expenditure of fixed assets 11. Therefore, we can track medical expenditures accurately. The direct costs of treating cancer include doctor visits, laboratory tests, imaging tests, radiation treatments, hospital stays, drug costs, etc. 12. For cancer patients, the insurance premium levels 13, hospital level 14, length of stay 15 and surgery 16 all affect medical expenses.

Therefore, it is crucial to gain a population-level view of the expenditures for lung cancer from the perspective of sociodemographic characteristics and the distribution of treatment cost characteristics for health care decision-making. Considering the aforementioned literature, both surgery and length of stay have demonstrated an association with hospitalization expenses in lung cancer patients. However, there is limited information to explain the association between the other contributing factors and hospitalization expense in lung cancer patients. Thus, we aimed to investigate the mediating effect of surgery and length of stay on the association between these variables and hospitalization expense among lung cancer patients. We hypothesized that surgery would be associated with a high risk of hospitalization expense through an extended length of stay in the hospital. Moreover, better insurance status would be associated with a high risk of hospitalization expense through active treatment (e.g., surgery).

Currently, China is reforming its national health strategy and health care system. Policies and interventions for a more equitable health system are being implemented in this period 17. An assessment and describing traits of the cost of treatment in China is a necessary first step in achieving this procedure. Therefore, analyzing the influencing factors of the current curative expenditure (CCE) in patients with lung cancer is essential. The data calculated based on the SHA 2011 framework may help determine the decisions linked to the allocation of resources, estimate the monetary flows related to curative care expenditure, analyze the probable factors that influence the CCE, protect the rights of patients and achieve the universal health coverage strategy that was proposed by the WHO.

## Methods

### Data sources

The prevalence of lung cancer in Hunan Province is similar to that in other provinces of China 18. This population-based study selected the patients in Hunan Province as a sample population, and the macrodata and demographic data were derived from work documents at the national and provincial level. The health expenditure was extracted from the Hunan Health Statistical Yearbook (2016), Hunan Health Financial Yearbook (2016), Hunan Health Report (2016), medical institution records and public health institution records.

### Quality control and data management

The process of gathering data was classified and coded according to the International Classification of Disease, the 10th revision (ICD-10). The records of lung cancer patients were coded as C34 in the ICD-10. The data extraction, audits, cleaning and calculations were maintained by implementing the basic accounting guidelines of the SHA 2011 framework 19 and a series of training courses. The staff involved in the data cleaning procedures were trained by the National Health Commission of China and evaluated through an examination. All data were entered electronically into a data terminal that was directly connected with Stata 10.0 (Stata Corporation, College Station, TX, USA).

### Study sample

Multistage stratified probability-proportional-to-size random sampling method was used to ensure that there was a representative sample. The sampling proportion was 1/3 for each stratum. Randomization was achieved through lottery-style drawings (prefecture-level cities), or a computer program (selection of streets, communities and towns). A stepwise approach was used for the whole sample extraction process, with several stages described below. The first stage was to select sample cities from Hunan Province based on a comprehensive consideration of geographical location, levels of economic development, the state of medical care and the health information management system. Thus, the 5 cities of Changsha, Yueyang, Zhuzhou, Yongzhou and Hengyang were selected. In the second stage, one district and three counties (county-level cities) were randomly selected as prefecture-level cities. Within the selected districts and counties, 5-8 streets, communities and towns were randomly selected, and 2-6 villages were randomly selected from each township as sample units. After determining the sample area and sample size, the third stage was to randomly classify the samples according to the type of medical institution and the administrative level of the health care institutions and professional public health institutions. A total of 1,072 medical institutions with 71 hospitals, 27 public health institutions, 131 community health service centers or township hospitals, 678 village clinics and 165 clinics were enrolled. The basic information was collected from the information system of the institute (Outpatient and inpatient care data were enrolled), including age, sex, disease, expense, date of hospital visit, length of stay and financing scheme.

The quality of the submitted data was checked and evaluated by using a tool to sort the cases and identify the missing and/or unexpected values, outliers and suspected errors. Then, the incomplete data were confirmed in the original health institution to ensure that they were correct. Finally, 2011 items that could not be amended were abandoned. A total of 46,214 valid items for lung cancer from January 1 to December 31, 2016 were collected. The data included the new lung cancer patients in 2016 and the patients diagnosed before 2016 but still treated in 2016. A standardized basic database was established.

### Descriptive statistics of the CCE for lung cancer based on the SHA 2011 framework

Medical service expenditure, government subsidies, and basic expenditure subsidies were included in the costs of lung cancer. These data were from Hunan Health Statistical Yearbook (2016), Hunan Health Financial Yearbook (2016), and Hunan Health Report (2016). The concrete calculation method was previously published and can be seen in the reference article 11.

### Analyses of influencing factors of hospitalization curative care expenditure for lung cancer patients

A total of 9,008 items of inpatient data was extracted from the whole 46,214 valid items of lung cancer. Spearman's Rank-Correlation test was performed to analyze the correlations between hospitalization expenses, surgery, length of stay, insurance status, institution level, sex and age. The hospitalization expenditure was not normally distributed, and the data were normally distributed after a logarithmic transition. Multiple stepwise regression analyses were used. A path analysis was performed by structural equation modeling to test the significance of the associations and to identify the direct and indirect effects of the variables.

The statistical software SPSS 21.0 (SPSS Inc., Chicago, IL, USA) and AMOS 20.0 (SPSS Inc., Chicago, IL, USA) were used for the analyses. The significance for all statistical tests was indicated by a two-tailed p-value of 0.05.

## Results

### CCE situation

Based on the SHA 2011 framework, the CCE for lung cancer was 8063.75 million CNY (1 USD ≈ 6.64 RMB, 2014), which accounted for 0.74% of the total CCE in Hunan Province. Most of the CCE for lung cancer patients was in hospitals (general hospitals, special hospitals and traditional Chinese medicine hospitals), and the expenditure was 7743.52 million CNY, which accounted for 96.03% of the total CCE of lung cancer patients. This was followed by public health institutions, with an expenditure of 319.69 million CNY that accounted for 3.96% of the total CCE of lung cancer patients, and basic medical institutions, with an expenditure of 0.48 million CNY that accounted for 0.01% of the total CCE of lung cancer patients. Overall, the CCE of lung cancer patients was mostly distributed in hospitals instead of in other medical institutions.

**Figure 1:**
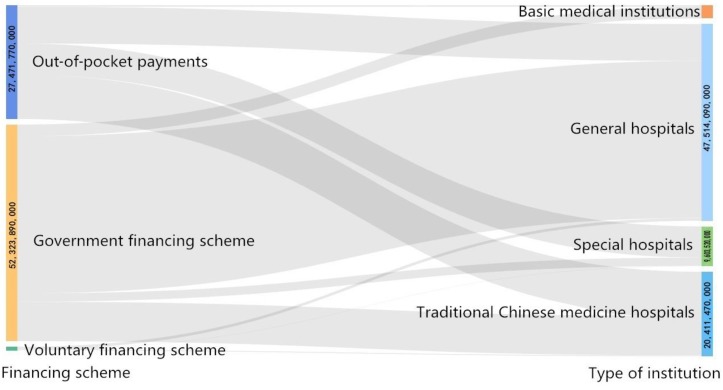
Flow of the CCE in different health institutions demonstrated by the Sankey diagram (CNY).

The financing scheme in Hunan Province was divided into the following three types: public financing scheme, voluntary financing scheme and family health expenditures (out-of-pocket payments (OOPs)). The OOPs for lung cancer patients were 2741.78 million CNY, which accounted for 34.00% of the total CCE of lung cancer patients. The three financing schemes were all mainly allocated towards inpatients (Table 1). In different types of hospitals, the financing scheme had variable distributions. The lung cancer CCE flowed 4747.87 million CNY to general hospitals, which accounted for 58.88% of the total CCE; traditional Chinese medicine hospitals received 2035.79 million CNY, which accounted for 25.25% of the total CCE; and special hospitals received 959.87 million CNY, which accounted for 11.90% of the total CCE (Figure 1).

Variations in the CCE of lung cancer patients existed not only across types of hospitals but also across medical institution levels. The CCE of lung cancer patients flowed 2696.14 million CNY to provincial medical institutions, which accounted for 33.44% of the total CCE; 2605.25 million CNY to municipal medical institutions, which accounted for 32.31% of the total CCE; 2442.13 million CNY to district and county medical institutions, which accounted for 30.29% of the total CCE.

### Composition of the CCE among lung cancer patients

**Figure 2:**
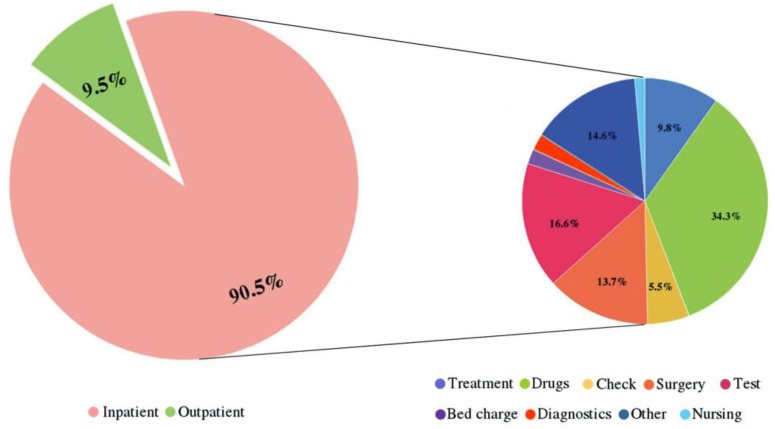
Situations that led to outpatient and inpatient expenses and the proportion of expenditure for each hospitalization expense item.

**Figure 3:**
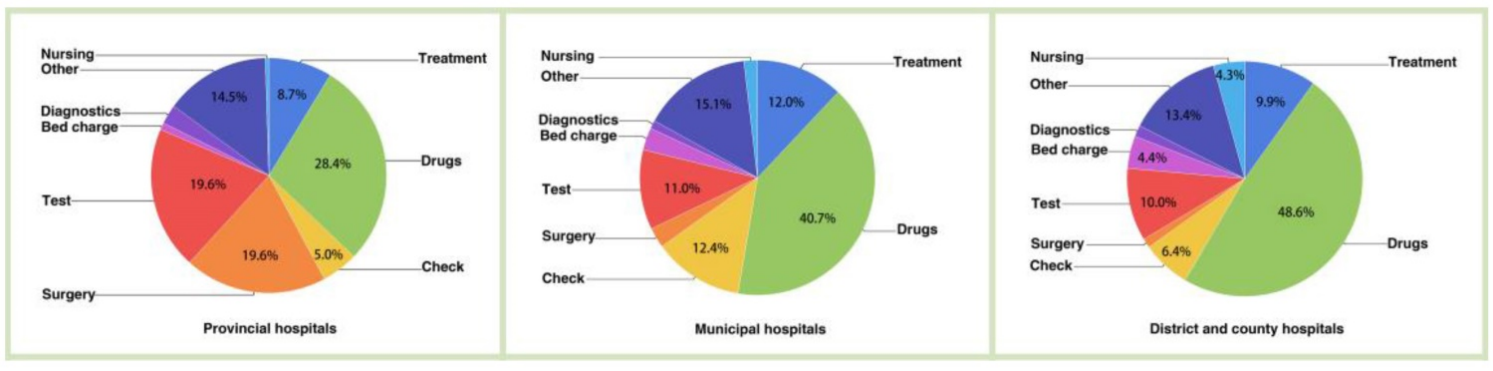
Proportion of expenditure items by hospital level.

**Figure 4:**
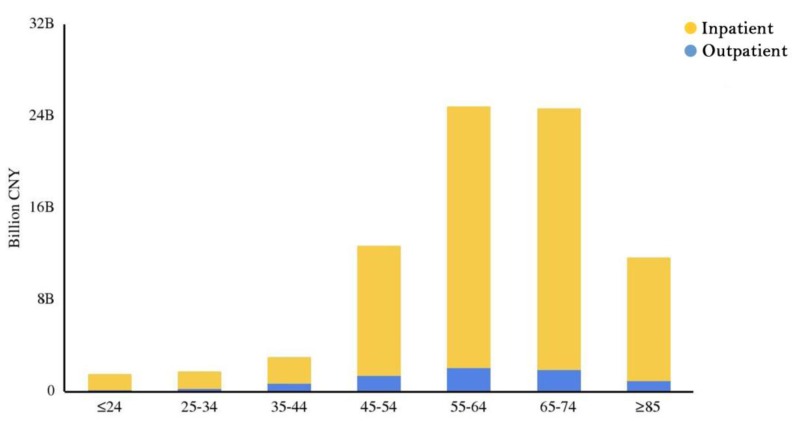
Distribution of outpatient and inpatient expenditures in different age groups. Influencing factors for hospitalization expenditures.

In general, the outpatient and inpatient CCE accounted for 9.49% and 90.51% of the CCE, respectively. That is, the inpatient expenditure was nearly 9.5 times greater than the outpatient expenditure. In addition, for lung cancer patients in hospitals, drugs, tests and surgery were the major aspects of the CCE (Figure 2). Figure 3 clearly showed that the aspects of induced CCE among lung cancer patients were dramatically different in different hospital levels. For example, the constituent ratio of drug costs was lower in provincial hospitals (28.40%) than in district and county hospitals (48.57%). Analyzing the CCE by age reveals that a significant portion of the expenditure occurred in patients aged 55-74 y, accounting for nearly 61.58% of the CCE (Figure 4). Thus, the medical expenses of elderly lung cancer patients were responsible for the increase in CCE.

### Factors correlated with hospitalization expenditures

A correlation analysis was conducted to find the intercorrelations among study variables. The results of Spearman's rank correlations were shown in Table 2. In particular, the hospitalization expenses correlated significantly and positively with surgery (rs = 0.481, p < 0.001), length of stay (rs = 0.668, p < 0.001), insurance status (rs = 0.325, p < 0.001), institution level (rs = 0.585, p < 0.001) and sex (rs = 0.033, p < 0.001). The hospitalization expenses were negatively correlated with age (rs = -0.041, p < 0.001). Age was also negatively correlated with both insurance status and institution level (rs = -0.073 and -0.085, respectively, p < 0.001). Lung cancer patients with better insurance were correlated with aggressive surgery (rs = 0.262, p < 0.001). Similarly, surgery was also related to an extended length of stay (rs = 0.232, p < 0.001).

### Major factors that affect hospitalization expenditure

A multiple stepwise regression analysis established a regression equation. This regression equation was significant (F = 1526.550, p < 0.001) (Table 3). As the type of hospital was a 3-level unordered nominal variable, two dummy variables (GH and SH) were constructed. The correlation analyses, tolerance and variance inflation factors were included to analyze the multicollinearity among study variables and revealed weak multiple collinearity among them. From the standardized regression coefficient, the top three factors influencing logarithmic hospitalization expenditure were surgery, type of hospital and insurance status. Length of stay, institution level, and sex were also included in the regression equation. While age was excluded B = 3.094, β = 0.002, t = 0.260 and p = 0.795. All the independent variables that were incorporated into the equation accounted for 57.5% of the degree of variation in the dependent variables.

### Testing the mediational model

**Figure 5:**
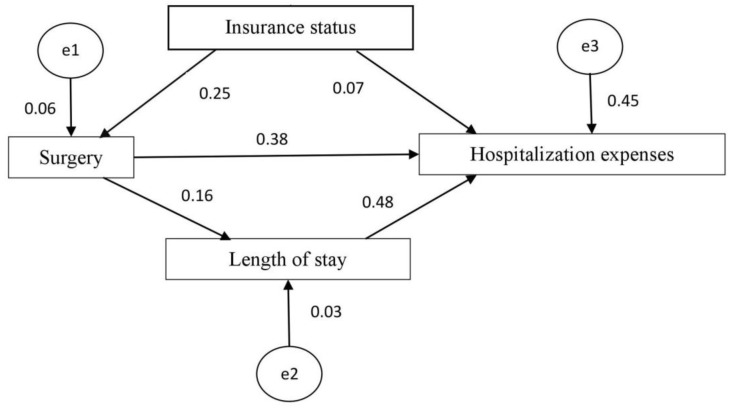
Path diagram of the structural equation model.

We conducted subsequent path analysis with structural equation modeling. The multicollinearity variables or variables independent of hospitalization costs were excluded from the subsequent mediational analyses 20. We found a statistically significant mediating effect of surgery on the association between insurance type and hospitalization expenses. There was also a statistically significant mediating effect of length of stay on the association between surgery and hospitalization expenses. The results of the structural equation modeling are displayed in Figure 5. According to the goodness-of-fit indices 21, the measurement model provided a reasonable fit to the data (χ2 = 1.373, p = 0.241 > 0.05) (Table 4). The results of the estimated model are shown in Figure 5 and Table 5 and demonstrate the standardized path coefficients of the significant structural relationships (p < 0.05) among the tested variables. Overall, insurance status, surgery and length of stay can explain 45% of the variability in hospitalization expenses.

## Discussion

### CCE of lung cancer patients

In this study, lung cancer CCE was calculated under the SHA 2011 framework. SHA 2011 was suggested to be used to produce National Health Accounts by all of the countries in the world, which makes the calculated result comparable across countries. Therefore, these results can be applied for comparisons among various countries. This study found that lung cancer accounted for a large proportion of health resources, with a total of 8063.75 million CNY in Hunan Province in 2016. When the CCE of lung cancer patients by age group were compared, the CCEs were found to be diverse between different age groups. The patients who spent the most were mainly aged 55-74 y. The CCE patterns by age closely followed the lung cancer incidence patterns. The median age at lung cancer diagnosis was approximately 70 y, with approximately 10% of cases occurring under age 55, 53% occurring between age 55-74 and 37% occurring over age 75 22. It is worth noting that, although the lung cancer patients aged 45-54 y did not constitute a large proportion of all lung cancer patients, their CCE was high. This result is consistent with a previous study that showed that the cost of cancer treatment is higher in younger patients than in older patients 23. This finding may not reflect the real demand for medical resources. Younger cancer patients are more likely to seek aggressive surgery and adjuvant therapy than older patients 24. The proportion of nontreated patients also increased with age 25. Economic factors played a role. Younger lung cancer patients were inclined to have better economic status, and most younger patients tended to go to higher-level hospitals and use higher-priced services than older patients. Unfortunately, the high expenditure for curative treatments may be a barrier for lung cancer patients with poor economic status. Therefore, it is time to revisit the medical care needs of different lung cancer patients and develop insurance benefit programs to promote the efficient use of medical services.

In this study, the OOP rate in the CCE was 34.00%. This rate is close to what was reported by Chunhai T et al.: the share of OOPs accounted for 29.97% of the total health expenditure 26. Although there is still a large gap in OOPs between China and some developed countries, the gap has narrowed. The goal of lower OOPs has already been scaled up at a national level. To improve the accessibility of medical care, the Chinese government has invested considerably and made many efforts to improve the financial protections provided by insurance. According to a report by the Organization for Economic Cooperation and Development and the World Health Organization, of all the countries in the Asia-Pacific region, China's out-of-pocket medical expenses have dropped the most 27.

The expenditure of each item contributed to CCE growth. Regarding the expenditure list among different hospital levels, drugs were the major driving contribution to the CCE. This finding is consistent with a previous report 28. Chemotherapy is one of the main treatments for lung cancer 29. New drugs have been developed to treat lung cancer, which has greatly increased the cost of treatment 30. From this study, as the hospital level declines, the proportions of both surgery and tests also decline. For cancer patients, tumor diagnosis is an important factor for therapy choice 31. To obtain a reliable diagnosis and undergo successful surgery, most patients intend to go to high-level hospitals.

### Distribution of the CCE in medical institutions for patients with lung cancer

It is observed that much of the lung cancer patients' CCE was spent in hospitals instead of in other types of medical institutions. In terms of the CCE distributed across different levels of medical institutions, more of the CCE was spent in high-level institutions than in low-level institutions. Our study also found that the CCE varied in different hospital types, mainly in general hospitals. This finding may be because medical resources are distributed unevenly in China. The type of medical institution is closely related to its diagnostic capabilities, treatment level, medical technology, level of care, etc. With the acceleration of urbanization and the lagging supply capacity of grassroots health facilities in China, high-quality medical resources are concentrated in large hospitals, and primary medical institutions are seriously lacking in medical resources 32. The medical facilities in grade III, class A hospitals are much better than those in other small hospitals, and people prefer to go to the relatively high-level hospitals for treatment 33. Meanwhile, a treatment mode of “minor illness in the community, serious illness to the hospital, rehabilitation back to the community” has been established in China. Patients with severe diseases tend to go to hospitals with higher levels of treatment 34. As cancer is considered a serious disease to every patient, patients are inclined to go to high-level and authoritative medical institutions to seek reliable therapies.

### Factors influencing CCE

In total, 96.03% of the CCE for lung cancer patients was in the hospital, and 90.51% of the hospital expenditure was inpatient expenditure. Thus, a focus on the analysis of hospitalization expenses is necessary. Describe the traits of lung cancer patients' curative expenditure constitutes an essential first step in clarifying the treatment relating factors, providing data-driven evidence to key stakeholders, and achieving rationalization of the utilization of health resources for the treatment of patients. The study on the influencing factors of the CCE in China can provide a basis for the rational allocation of limited health resources. It is not only the core direction of the field of health economics but also in line with the development trend of proper individualized treatment of lung cancer. We found that the relationships between study variables and hospitalization expenses were significant, including the length of stay, surgery, type of hospital, insurance status, institution level, age and sex. Notably, these findings are in line with those of other research 35, 36.

Surgery had an inevitable effect on lung cancer patients. Surgery not only is associated with increased cost 37 but also necessitates the extension of the hospitalization duration 38. Our findings indicated that length of stay mediated the relationship between surgery and hospitalization expense for lung cancer patients, demonstrating that the length of stay played a role in buffering the adverse effects of surgery on hospitalization expense. The length of stay is often considered a key indicator of medical resource consumption 39. The longer the length of stay is, the more medical resources will be consumed, which induces higher hospitalization expenditures 40. In other words, shortening the length of stay is an effective way to reduce the CCE in patients with lung cancer.

In this study, we found that insurance status is a major variable that can affect both surgery and hospitalization expenses. This finding may be attributed to several factors. For patients who have medical insurance, the medical expenditures were paid partly by the insurers, so the financial burden of lung cancer patients and their families may be relatively small. Although patients with medical insurance were prone to have a high CCE, the copay portion of the treatment costs was reduced, and the absolute quantity of expenditure was lowered, so these patients were more likely to take aggressive measures, such as surgery. The financial consequences of cancer may interfere with receiving health care services 41. Patients with better economic conditions are more likely to pay for certain health care services 42. Although the basic purpose of medical insurance is to apportion the economic risk of individual illness and promote fairness 43, there are still large gaps between different types of medical insurance in China. Notably, according to our survey, some lung cancer patients still have no medical insurance, and their CCE was paid entirely out of pocket. A study investigating the financial burdens of cancer patients showed that 94.2% of cancer patients at the diagnosis stage and 86.2% of cancer patients at six months after diagnosis found their financial burden to be at least somewhat difficult to handle 44. Thus, we can surmise that the plight and financial burden of lung cancer patients without medical insurance in China are worse than those who have medical insurance. One of the core goals of universal health coverage is to avoid large individual health expenditures and catastrophic health expenditures 45. Since 1997, the Chinese government has committed to developing social medical insurance. Recently, more efforts have been made to provide better health care 46. However, China is a developing country with a large population, and there are still many steps required to promote health insurance coverage. Meanwhile, in this study, although the CCE of insured cancer patients was reduced by insurance, the overall ratio of OOPs for patients with lung cancer was still high, and further financial support remains necessary.

Age was negatively correlated with the hospitalization expenditure of lung cancer patients. As this association was not robust and disappeared after regression analysis, we cannot exclude the secondary effects that are attributable to the relationship of other factors. Empirical evidence suggested that the population age had only a modest effect on health expenditure growth 47. Similarly, this study showed that age was not an important driver of the expenditure in lung cancer patients. However, in the long term, the increased longevity could gradually postpone health expenditure from one age group to the next 48.

Although previous studies have suggested the role of some factors in affecting the cost of lung cancer, no research had verified the structure-effect relationship using proper statistical models in lung cancer. On this basis, this study used the structural equation model to probe the specific influence degree and the interrelationship of these variables and achieve better explanatory efficacy. 45% of the variance of the extent of hospitalization expenses explained by the study factors. It means these factors highly determined the hospitalization expenses outcome. From this study, in addition to patient characteristics and treatment factors studied, variables more likely to improve the degree of determining hospitalization costs may be beyond the scope of this manuscript but will be worthily explored in future research. In the context of precision medicine, breakthroughs continue to be achieved in treating lung cancer. Molecular targeted therapy, immunotherapy, and stereotactic body radiation therapy were verified as better treatment strategies in certain pathological types and some disease stages of lung cancer patients 49, 50. Therefore, in the future, we can refine the costs of different treatment methods and the types of patients, to provide more specific guidance for practice, which is also the future development trend.

Limitations should be considered in interpreting the results of the study. First, the cross-sectional nature and the “hit and miss” facet generated from random sampling of institutions might cause some selection biases. Second, most lung cancer patients are middle-aged or elderly people, and they may have other diseases at the same time. In the course of disease treatment, the cost recorded may include the treatment expense of other comorbidities besides lung cancer.

## Conclusion

In summary, the application of the SHA 2011 framework promoted the way that CCE was interpreted. Under this framework, the constituent ratio of CCE, the status of insurance schemes, and financing needs can be interpreted. Health care coverage and access to health care services are still issues for lung cancer patients in China. The model used in this study provided an articulate description of the direct and mediation effects related to hospitalization expenses of lung cancer patients. When interpreting the CCE for patients with lung cancer, close attention must be paid to the contributing variables (insurance status, surgery, and length of stay) themselves and the interrelationship among them. These results highlight the need to both account for and potentially reduce the total CCE among high-incidence populations in obtaining cancer care.

## Figures and Tables

**Table 1:** Allocation of the financing scheme for inpatients and outpatients (million CNY)

Service function	Public financing scheme		Voluntary financing scheme		OOPs
	Social health insurance	Government financing scheme	Nonprofit organization financing	Enterprise financing plan	Family health expenditure
Outpatient	124.07	52.00	0.00	2.95	586.61
Inpatient	4475.67	580.69	30.50	56.05	2155.17

**Table 2:** The relationship among hospitalization expenses, surgery, length of stay, insurance status, institution level, sex and age of lung cancer inpatients (*r_s_*)

Variables		1	2	3	4	5	6
1	Hospitalization expenses	1					
2	Surgery	0.481**	1				
3	Length of stay	0.688**	0.232**	1			
4	Insurance status	0.325**	0.262**	0.092**	1		
5	Institution level	0.585**	0.542**	0.176**	0.375**	1	
6	Sex	0.033**	-0.023*	0.008	-0.004	-0.007	1
7	Age	-0.041**	-0.011	0.003	-0.073**	-0.085**	0.075**

Abbreviations: *r_s_* =Spearman's correlation coefficient. * p < 0.05, ** p < 0.01. Note: Surgery, insurance status, institution level and sex were coded as follows. For surgery: no surgery = 0 and surgery = 1. For insurance status (sorted by reimbursement ratio): self-funded = 1, new rural cooperative medical care or urban and rural medical insurance = 2, urban residents' basic medical insurance = 3 and urban employees' basic medical insurance = 4. For institution level: district and county hospital = 1, municipal hospital = 2 and provincial hospital = 3. For sex: female = 1 and male = 2.

**Table 3:** Multiple stepwise regression analysis using logarithmic hospitalization expenses of lung cancer patients as a dependent variable

Variables	Unstandardized coefficients		Standardized coefficients	*t*	*p*-value
	*B*	Standard Error	*β*		
Constant	-6669.021	1112.946		-5.992	<0.001
Type of hospital					
GH	2449.689	334.497	0.066	7.323	<0.001
SH	19612.994	424.750	0.386	46.175	<0.001
Length of stay	752.051	11.070	0.473	67.938	<0.001
Surgery	18262.116	407.652	0.386	44.798	<0.001
Insurance status	1206.554	152.728	0.061	7.900	<0.001
Sex	783.360	290.439	0.019	2.697	<0.001
Institution level	-572.175	231.621	-0.026	-2.470	0.014

Model fit: F = 1526.550, p < 0.001; R^2^ = 0.575; adjusted R^2^ = 0.575. Note: Two dummy variables were needed to represent the types of hospital which had three unordered levels (general hospital, traditional Chinese medicine hospital, and special hospital). They were abbreviated and constructed as follows: GH=General hospital= "1" if it was a general hospital and "0" otherwise; SH= Special hospital = "1" if it was a special hospital and "0" otherwise. As for the traditional Chinese medicine hospital, setting both GH and SH to "0" indicated it was a traditional Chinese medicine hospital.

**Table 4:** Fit indices for the tested model

Item	χ^2^/*df*	GFI	AGFI	CFI	NFI	IFI	TLI	RMSEA
Model value	1.373	1.000	0.999	1.000	1.000	1.000	1.000	0.006
Recommended value	1<χ^2^/*df*< 3	>0.90	>0.90	>0.90	>0.90	>0.90	>0.90	<0.08

**Table 5:** Indirect effects of insurance type and surgery on hospitalization expenses

Model pathways	Unstandardized coefficient	
	Indirect effect	Total effect
Insurance status→Surgery→Hospitalization expenses	0.095	0.165
Surgery→Length of stay→Hospitalization expenses	0.079	0.457

## Abbreviations

CCE: current curative expenditure
SHA 2011: System of Health Accounts 2011
CNY: Chinese Yuan
ICD-10: International Classification of Disease, the 10th revision
OOPs: out-of-pocket payments
GH: general hospital
SH: special hospital
